# From Bank Preparation to Clinical Use of Homologous Skin Allografts in Wound Healing: A Sustainable Approach

**DOI:** 10.3390/life14101285

**Published:** 2024-10-11

**Authors:** Laura Amoroso, Serena Agueci, Elisa Pianigiani, Francesca Ierardi, Laura Calabrese, Pietro Rubegni, Linda Tognetti

**Affiliations:** 1 Skin Bank Unit, Azienda Ospedaliera Universitaria Senese (AOUS), 53100 Siena, Italy; 2 Dermatology Unit, Department of Medical, Surgical and Neurosciences, University of Siena, 53100 Siena, Italy

**Keywords:** homologous skin allograft, allografting, skin bank, immunohistochemistry, histology, MTT assay, wounds, ulcers, hard-to-heal wounds, practical application

## Abstract

Given progressive population ageing and the increase in the number of patients with comorbidities, the management of chronic and/or hard-to-heal wounds (HHWs) nowadays represents a common problem in many clinical settings. In these cases, standard strategies may not be sufficient. Autologous grafting represent the gold standard for permanent wound closure, but is almost never realized when the skin loss is extensive/the patient is young. The grafting of homologous skin/dermal tissue procured from cadaver donors (i.e., allografting) represents the best alternative, especially when the dermal component is lost. This request supports the activities of skin bank establishments (including donor screening, skin procurement, processing, storage, and distribution) that are regulated by specific guidelines and need to continuously meet quality standard requirements. The aim of this work is to both give specific insights of all the procedures implied in allograft preparation as well as an overview of their practical application in the treatment of different HHWs. The particular characteristics of each skin/dermal allograft released by Siena Skin Bank (cryopreserved/glycerol-preserved skin/de-epidermized dermis, acellular lyophilized de-epidermized dermis/reticular dermis) are also discussed. The exemplificative series of HHWs managed in the Dermatology Department of Siena were classified according their etiology into post-traumatic, vascular (arterial/venous/mixed/lymphatic), inflammatory, surgical, and heat/chemical burns. Globally, the clinical advantages obtained include: acceleration of healing process, pain sparing, resistance to bacterial contamination, dermal regeneration (instead of scarring), and better aesthetic–functional outcome.

## 1. Introduction

The skin plays multiple fundamental roles in the correct preservation of the physiology of the human body, including thermal regulation, fluid balance, protection from infections, and resistance to external mechanical forces [1]. Indeed, the skin acts as an effective physical, chemical, and bacterial barrier. Although the gold standard for permanent wound closure is autologous grafting (i.e., autografting) [2,3], this procedure cannot be realized in most cases, where the skin loss is extensive/the patient is young. As an alternative to autologous grafting/autograft, homologous skin/dermal grafts (i.e., allografts) procured from cadaver donors can be regarded as the best treatment for burned patients, being the most “physiological” skin substitute in wounds of various origin [4,5]. Allografts differ from xenografts, which are sterilized and processed tissue derived from an animal (generally a cow or pig).

Homologous skin and dermal grafts/allografts have been used in the last 50 years in many wounds of different etiology and morphology because of the relevant clinical benefits, including: (i) promotion of re-epithelization; (ii) shortening of healing time; (iii) alleviation of pain; and (iv) protection of deep subcutaneous structures including tendons, bones, cartilage and nerves [6].

A wide variety of skin and dermal equivalents is currently available on the market, including fully synthetic and semi-synthetic ones (i.e., one synthetic structure combined with one/two layers of cultured cells): there are now more than 75 skin substitutes, and the number is still rising [1,2,3,4]. Nevertheless, viable human skin allografts are still considered the best solution for large, deep burns and hard-to-heal wounds (HHWs), where human skin/dermal allografts demonstrated significantly better clinical outcomes [6,7]. Given progressive population ageing and the increase in the number of patients with comorbidities, the management of HHWs nowadays is a common problem in many clinical settings. Indeed, those cases usually do not respond to the standard of care and need advanced dressing/integrated approach, e.g., skin allografting [1,2,3,4,5,6,7].

To avoid any concern about the potential risk of transmission of pathogens with allografting, especially in immunocompromised patients, extremely thorough cadaver donor selection based on medical records and serological screening must be performed [8,9,10,11,12]. All processing steps, from procurement to data recording, processing, and storage, must follow written procedures, drafted in accordance with national laws and technical guidelines [13,14,15].

In this paper, we aimed to: (i) describe in detail all the procedures related to skin allografts starting from procurement to banking, processing in the Siena Skin Bank, and distribution in the Tuscany/Italian territory; (ii) characterize if/how the different preservation methods (freezing/glycerolizing/freeze-drying) may impact the tissue morphology and structure; and (iii) give an overview on the use of different skin allograft produced in Siena Skin Bank in a series of exemplificative HHWs of different etiology.

## 2. Materials and Methods

### 2.1. Ethics

This retrospective monocentric study was conducted in accordance with the Declaration of Helsinki. Informed patient consent for anonymized image reproduction of the skin lesions was obtained. All data were anonymized before use. Ethical committee approval was waived, as the data were collected as part of routine activity and then retrospectively analyzed.

### 2.2. Siena Skin Bank

The skin bank of Siena was established in 1999 in the Dermatology Department of Santa Maria alle Scotte Hospital [8] with the purpose of selecting, preserving, and distributing human tissue grafts procured from cadaveric donors for transplantation, in full compliance with statutory process (D.Lgs. 191/2007, D.Lgs. 16/2010, EU Guidelines to Good Manufacturing Practice (GMP) Annex 1 [13]), following European (EDQM Guidelines 2022) [14] and Italian (CNT guidelines 2016) [15] regulations, in accordance with specific product quality standards (ISO) [16,17,18,19]. Procurement is performed 24 h/day by a team composed of medical practitioners and nurses, officially authorized after a full training course (i.e., nurses operate under medical responsibility). In a surgical theater, and under aseptic conditions, skin tissue is collected from the donor’s posterior trunk and lower limbs by using a battery-operated dermatome, after appropriate disinfection of the skin surface with povidone-iodine scrub and chlorhexidine solutions. The tissue is then transported to the Skin Bank in refrigerated tanks (+2 °C/+10 °C). All the processing procedures are performed in laboratory cleanrooms containing GMP grade A laminar flow cabinets, with a grade B background, in accordance to Skin Bank procedures [13] and guidelines [14,15,16,17,18,19].

### 2.3. Donor Selection

To minimize the overall risk of infection, it is essential that trained personnel evaluate the eligibility of donors prior to skin donation, through physical inspection, accurate assessment of clinical records, and appropriate serological screening. Exclusion and inclusion criteria for skin donation are clearly set in the regulations and guidelines [14,15] and must be evaluated in all donors.

The objective of laboratory assessments is to screen donors for human immunodeficiency virus (HIV 1 and 2), major hepatitis viruses (HBV–HCV), Treponema pallidum (TP), and cytomegalovirus (CMV) by examining circulating blood antigens and corresponding antibody responses. Biomolecular tests for HIV–HBV–HCV are performed by PCR or NAT on donor blood samples, as the quickest approach [12]. This methodological approach, although expensive, has proven particularly effective, as it has enabled our laboratory to distribute, as of 31 December 2023, more than 500,000 cm^2^ of skin from 2650 donors for a total of 24,160 grafts, without infection transmission. In addition, strict quality control during tissue processing, banking, and storage is required.

### 2.4. Skin Bank Tissue Grafts

**Figure 1:**
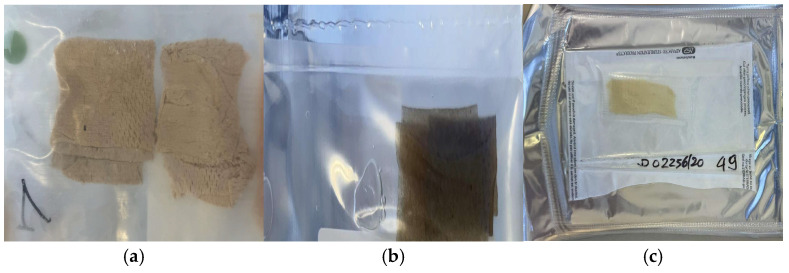
(**a**) Cryopreserved skin; (**b**) glycerol-preserved skin; (**c**) γ-irradiated acellular DED.

The demand for skin allografts currently arriving at the Bank comes from burn units and the general surgery, oncologic surgery, vascular surgery, plastic surgery, odontostomatology, and maxillofacial surgery units, as well as from the dermato-surgery and vulnology units. Aiming to respond to this demand, based on the characteristics of the procured tissue and the intended clinical application, the grafts undergo different preservation processes: cryopreservation, glycerol preservation, and lyophilization/freeze-drying (Figure 1).

#### 2.4.1. Cryopreservation

Skin tissue (epidermis and dermis) undergoes a disinfection process by consecutive washing steps in alcohol and saline solution enriched with penicillin and streptomycin before being incubated overnight in a cryoprotective solution (Base_Alchimia with 15% glycerol) previously validated by the skin bank [20]. On the following day, the skin is frozen using a controlled-rate freezer (at −1 to −2 °C per minute, from +10 to −90 °C Planer Kryosave Integra, mod. 750) and then stored at −80 °C for up to two years. The cryopreserved tissue maintains its osmotic, structural, and mechanical properties, along with a certain level of cellular vitality; metabolically active skin grafts are generally preferred by surgeons due to the graft’s ability to stimulate rapid neovascularization of the wound bed and eventually become integrated, i.e., in severe burns or epidermolytic diseases [20,21,22,23,24,25,26].

#### 2.4.2. Glycerol Preservation

The tissue is incubated with solutions containing physiological saline and glycerol at increasing concentrations (50%, 70%, and 87%) and then packaged and stored at +2/+10 °C for up to five years. In this case, the tissue is not viable but maintains its mechanical and structural properties. This biological dressing is particularly well-suited for the treatment of ulcers (pressure/post-traumatic and venous) due to its analgesic and antimicrobial properties, or for managing patients affected by Lyell’s syndrome [27,28,29].

#### 2.4.3. Decellularization, Freeze-Drying, and Gamma Irradiation of Dermal Matrices

Dermal allografts are obtained either from de-epidermization of skin (de-epidermized dermis (DED)) or from second passage of the excising dermatome reticular dermis (DER).

To obtain de-epidermized dermis (DED), the overlying epidermis is mechanically removed after overnight incubation in a saline solution. Both DED and DER can be further decellularized (patented procedure). The decellularization process involves four consecutive phases: cell lysis (incubation of the tissue in bidistilled water in a rocking incubator), membrane removal (through sonication of the tissue positioned in bags containing zwitterionic detergent of the bile salt class), detergent removal (through sonication within pouches containing a sodium phosphate monobasic buffer solution capable of buffering the acidity of the detergent without altering the structural properties of the tissue), and cryopreservation and packaging. Finally, strips are freeze-dried, gamma-irradiated at 25 kGy, and stored at room temperature. After adequate re-hydration in saline solution, the dermal acellular grafts regain their particular mechanical and structural properties [30,31,32,33,34]. The use of homologous acellular dermal matrices is particularly indicated in the treatment of “difficult wounds” with exposure of fragile structures such as bones and tendons, in the presence of necrotic scabs, or in debilitating clinical conditions such as diabetes or arterial/venous or lymphatic insufficiency [10,22]. In these cases, the use of homologous acellular dermal matrices allows tissue integration at the bottom of the wound, recolonization by donor cells, and stimulation of a new dermis with maintenance of physiological and functional properties, with a relevant reduction in re-epithelialization time [10,26,27,28]. Applications are various and include dermatological, plastic, oncological, general, bariatric, otorhinolaryngological, maxillofacial, dental, and orthopedic surgery [32,33,34,35,36,37].

### 2.5. Morphologic and Functional Tests

In order to verify that the tissues produced at the Skin Bank meet the required standards and are suitable for grafting onto patients, morpho-structural (histology and immunohistochemistry, on all tissue types) and functional (MTT, on cryopreserved skin) tests are routinely performed, in addition to microbiological and environmental (microbiological and particle) controls. To prove that tissue processing is carried out aseptically, it is necessary to verify that the working environment and the presence of the operators do not constitute a source of contamination. In particular, environmental microbiological control is carried out using an SAS-PBI air sampler (aspiration 1000 L/h), 90 mm diameter settle plates (during the process), and contact plates (at the end of each process), while operator contamination is monitored by glove print on 55 mm plates.

### 2.6. Cell Viability

**Figure 2:**
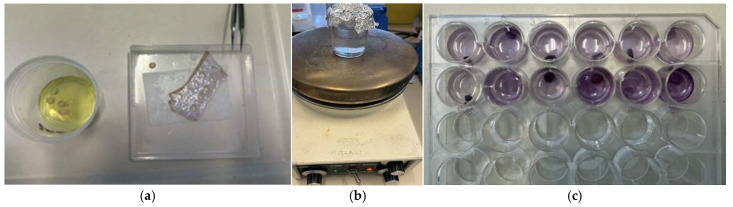
(**a**) MTT test phases. 6 mm ∅ punch biopsies. (**b**) Negative control obtained by boiling. (**c**) Multiwell plate for MTT test: cryopreserved tissue. Second row skin allograft before process; first row skin allograft after 15 days of storage at −80 °C.

The MTT (3-(4,5-dimethylthiazol-2-yl)-2,5-diphenyltetrazolium bromide) assay is a laboratory technique frequently used in most skin banks to assess cell viability because of its reproducibility, reliability, and rapidity of execution [20,21,22,23,24,25,26,27]. This assay detects the ability of mitochondrial enzymes, present in living cells, to transform tetrazolium salts (water-soluble) into formazan pigments (insoluble). These pigments are than dissolved, extracted by means of ethylene glycol (an organic solvent), and quantified using a spectrophotometer, which measures the optical density (OD) at a wavelength of 570 nm [20]. In cryopreserved tissues, the MTT test is used to determine cell viability before processing and after 15 days of storage at −80 °C. Specifically, for each allograft undergoing cryopreservation, five skin samples (6 mm ∅ punch biopsies) and one negative control obtained through boiling in distilled water for 30 min are analyzed (Figure 2).

By subtracting the OD value obtained for the negative control from the mean obtained for the five viable skin sections, the residual OD of the tissue can be obtained. Assuming that the OD obtained for the tissue before cryopreservation corresponds to 100% viability, it is possible to determine the residual viability of the tissue after 15 days of storage at −80 °C. In accordance with the guidelines of the European Directorate for the Quality of Medicine & HealthCare (EDQM), a cryopreserved tissue graft can be considered viable if a minimum of 20% of residual cell viability is reached [14].

### 2.7. Histology

To verify the maintenance of tissue architecture pre- and post-processing, all skin types processed at the Siena Skin Bank are subjected to histological analysis. Briefly: small sections of skin are fixed in formalin, dehydrated by passages in alcohol of increasing concentrations, diaphanized by passage in toluol, and embedded in paraffin. Finally, the 6-micron microsections obtained by microtome cutting are placed on slides and stained with hematoxylin–eosin.

### 2.8. Immunohistochemical Investigations

For immunohistochemical investigations, two antibodies have been selected to evaluate relevant structures to be verified in the cryopreservation, glycerol preservation and de-cellularization process, namely: anti-collagen IV (ab6586; Abcam (Cambridge, UK) and Ki 67 (La Roche, France, 790-4286) [32,33]. Indeed, collagen IV constitutes about 50% of the entire basement membrane (MB) [33], and it is found in the endothelium and epithelium of the glandular and pilar ducts, in the medium and deep dermis, and in blood and lymphatic vessels. By performing additional immunohistochemical assay, we aimed to morphologically detected the quantity of the residual IV collagen in tissues processed at our Skin Bank.

In particular, the MB (i.e., dermo-epidermal junction) is composed of a shiny lamina in contact with basal epidermal keratinocytes and a *dense lamina* in contact with the papillary dermis called the dense sublamina. Since the de-epidermalization process achieves dense sublamina cleavage, the Ki-67 antigen (clone MIB-1, dilution 1:100) is considered the most reliable proliferation index for the cell of the basal layer of the epidermis that may remain after de-epidermization [32]. Consequently, we also employed this additional staining assay.

### 2.9. Clinical Application of Skin Allografts in Wound-Healing

Human skin allografts/bank bioproducts bring several advantages in wound healing, such as significant control of pain and exudate, protection of subcutaneous structures (e.g., nerves, tendons, bones, cartilage), stimulation of functional new dermis production (rather than a scar), and re-epithelization with relevant reduction of wound closure time [37,38,39,40,41,42,43,44,45,46]. Thus, although a huge number of dermal matrices and skin equivalents are available on the market, either synthetic or semisynthetic, human skin allografts can still be considered the most physiological alternative to autologous skin in HHW management [37,38,39].

Wound closure after post-traumatic injuries and/or localized at particular body sites (head-and-neck, oral cavity, lower legs) is particularly challenging and can often be delayed due to local and systemic factors. In these cases, an integrated medical-surgical approach based on the use of dermal acellular matrices should be considered [39,40,41,42,43,44,45].

#### 2.9.1. Clinical Application Fields

Although the field of application of these bioproducts, in particular acellular dermal matrices, is likely to be implemented and possibly can be extended to almost all surgical/reconstructive fields, a series of application are nowadays validated and currently performed in our Siena hospital, including [39]: vascular ulcers, post-traumatic deep wounds, post-surgical dehiscence in the head, neck, trunk, and extremities, burns, deep fistulae, tendon repair, orthopedics procedures [39], maxilla-facial interventions (especially defect repair after skin cancer removal) [45], pyoderma gangrenousum management [46], post-mastectomy breast reconstruction [47], and otorinolaringoiatry reconstructions/tympanoplasty [48]. Moreover, there are favorable experience in the reconstruction of urethral, vaginal, and eyelid defects [39].

Usually, patients attending our *hard-to-heal wounds service* (outpatient clinic plus inpatient department) located in the Dermatology unit of Siena Hospital, are affected by: second- to third-degree burns (thermal, chemical, radiation, and electric), vascular ulcers (venous, mixed, or arteriosus), neoplastic ulcers, or inflammatory ulcers, alone (e.g., leukocytoclastic vasculitis, pyoderma gangrenosum) or in the context of connective tissue diseases (e.g., systemic sclerosis, localized scleroderma, Raynaud phenomenon), as well as dehiscence and post-surgical neoformation removal/surgical incision at various sites.

#### 2.9.2. Grafting Protocol

We previously described the particular benefits of the different skin allografts prepared in our skin bank [22,37] when applied to various type of wounds [38]. Briefly, all skin allograft clinical applications follow a three-step protocol [38], including: (i) adequate debridement, (ii) adequate wound bed preparation, and (iii) a reconstructive phase based on skin grafting.

The first step consists of wound bed debridement, which can be performed either mechanically (e.g., surgical knife/curette) or enzymatically (e.g., application of topical collagenase ointment/application of debriding dressings): the choice essentially depends on wound bed conditions at presentation time, patient sensitivity at the wound site (high VAS score), and availability of the proper environment for operating. In general, a thick eschar (i.e., in late presentation) is preferably surgically removed under local anesthesia, and large, extensive/critical HHWs may require rapid debridement and preparation by applying negative pressure wound therapy (NPWT). Indeed, this method is known to help removing adherent fibrin clots and contract wound edges, stimulating vital granulation tissue production and reducing the microbacterial burden. More recently, the use of low-frequency ultrasonic devices has proved to yield effective, rapid, and painless debridement.

At the second step, after proper wound bed preparation, the wound should be re-examined to select the optimal grafting type according depth, body location, margin type, wound bed structure, patient sensibility, etc. In general, the following techniques can be used: simple/composite grafts, meshed/non-meshed grafts, cryopreserved/deep- frozen/glycerol-preserved/lyophilized grafts, and sandwich technique/Cuono technique. [22,35,37,38]. The choice between a monolayer graft (i.e., one layer of skin or dermal graft) and a composite graft (i.e., dermal graft covered by skin allografts, to prevent desiccation) depends mainly on the wound extension. Limited HHWs (diameter < 7 cm, loss of substance limited to the deep dermis) may benefit from simple grafts, while extensive HHWs (diameter > 7 cm, loss of substance extended to deep subcutaneous structures with/without nerves/tendons/bone exposure) may require composite grafts. The third step consists of the application of skin bank bioproducts (either sutured or fixed with steri-strips onto the wound bed) and the dressing phase.

This three-step approach in clinical practice allows adequate management of HHWs, including full-thickness/critical skin loss [10,22,38]. In addition, we have to consider that acellular dermal grafts can protect deep exposed fragile structures and control local pain for a long time, as well as resist bacterial colonization, while being quickly colonized by the host cells and integrating into the wound bed. This globally reduces hospital admission and total healing time.

#### 2.9.3. Simple Skin Grafts

After reshaping the skin graft following the edges of the wound with simple surgical scissors, the single/monolayer/simple skin graft can be applied directly onto the wound bed. In general, an overlap of about 0.5 cm over the wound margin is recommended to ensure the stability of the graft at the wound site. On the other hand, in some cases, simple DED/dermis grafts can be applied in deep but small wounds avoiding overlap, after precise shaping: this dressing is limited to selected HHW cases, such as inflammatory/vascular wounds. In these cases, coverage is realized by means of multiple layers of paraffin gauzes applied with discrete compression [22,35,37,38]. The dressing can be re-evaluated after 7 days in case of low exudation: skin is generally removed after 7 to 10 days, as the adhesive properties are reduced and the new dermis formation below tends to reject the allograft. An exception is represented by burned skin due to local immunosuppression, where allograft skin can be partially or even almost fully integrated [23].

#### 2.9.4. Composite Skin Grafts (Sandwich Technique)

Composite grafts are constituted by two-layer grafting, i.e., the dermal graft is covered by a skin graft to avoid dermis desiccation/de-hydration. In any cutaneous HHWs, allografts should be covered by paraffin gauze and overlying moderately compressive cotton bandage (avoiding histolytic solutions like iodopovidone coming in contact with skin allografts). The dressing should be re-evaluated after 5 or 7 days according to exudate: during each evaluation, if the dermal graft is completely integrated onto the wound bed, a single graft is applied; if the dermal graft is partially integrated, resizing may be necessary. Skin graft of simple and composite dressing can be maintained at the site for 10 to 14 days. The skin is usually removed once the site has been sufficiently healed, or when an autologous graft is available for permanent wound coverage. Other uses of skin/dermal allografts include the reconstruction of donor sites (e.g., forearm/thigh/axilla/retroauricular area) for partial- or full-thickness autograft, respectively [22,35,37,38].

#### 2.9.5. Skin Grafts in Dermatologic-Plastic Surgery

The use of skin allografts from skin bank in dermatologic surgery is wide and varied and relies on the creativity of the dermo/plastic surgeon [14,21,43]. A frequent indication is represented by post-surgical wounds produced by skin cancer excision of the head and neck, areas where procurement of skin to realize flaps or autografts is challenging. Patients are indeed generally eligible in three cases: (1) critical body areas; (2) relevant co-morbidities; and (3) delayed Mohs surgery (temporary closure pending histological report). The grafting procedures are the same as described above, but a general preference for suture of the graft with resorbable stiches is observed, as well as the use of acellular dermis [22,38,39].

### 2.10. Clinical-Laboratory Correlates in Daily Practice

Identification of the most appropriate type of allograft for each HHW depends on etiology and clinical characteristics and may vary over time as the wound re-epithelialized, based on wound bed conditions.

We here aimed to illustrate some exemplificative applications of cryopreserved skin and to investigate whether a higher viability index correlates with a better clinical outcome.

Data from 13 consecutive patients attending the Siena Dermatology department between September 2023 and January 2024 were collected. They are the most representative cases of wounds and ulcers stratified per etiology: burns, surgical dehiscence, traumatic wounds, inflammatory wounds, and vascular wounds, which include arterial, venous, and mixed vascular wounds. All underwent simple or composite grafting with cryopreserved skin. The group was indeed recruited in a short period in order to have samples representative of real-life patients eligible for either simple or composite grafts with cryopreserved skin. Finally, one exemplificative case per etiology is discussed below in detail.

### 2.11. Statistical Analysis

Descriptive statistics (mean, range, and standard deviation for quantitative variables; absolute frequencies and percentages for qualitative variables) were applied to laboratory and clinical data.

## 3. Results

### 3.1. Viability Testing

Taking into account a total of 13 consecutive samples (from 2023), the average percentage of residual viability after 15 days of storage at −80 °C was found to be 50% (Table 1) and was comparable to the value obtained in our retrospective analysis from 26 samples performed in 2016 [20]. This confirms the validity of the cryopreservation method used.

### 3.2. Histologic Investigations

#### 3.2.1. Cryopreserved Tissue

**Figure 3:**
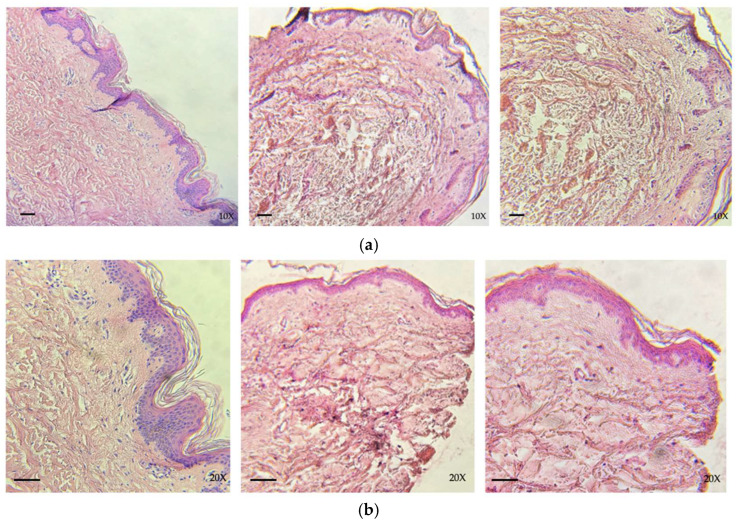
Histological evaluation of skin allograft samples before cryopreservation (**a**) and after 15 days of cryopreservation (**b**): the integrity of epidermal layer and dermo-epidermal junction is preserved (hematoxylin–eosin; scale bar: 50 µm; original magnification 10×, 20×).

Histological analysis performed on skin allografts destined for cryopreservation was performed over three consecutive samples (taken in 2023) and showed no morpho-structural changes resulting from the freezing process. The tissue did not show any degenerative-necrotic type phenomena, the stratum corneum was generally preserved, and the dermal-epidermal junction was intact, remaining substantially unaltered both before and after the process (Figure 3). In some cases, focal separation of the stratum corneum from the rest of the epidermis can be observed. However, this phenomenon does not seem to be related to freezing, but rather to the interval between tissue collection and storage if it exceeds 48 h.

#### 3.2.2. Glycerol-Preserved Tissue

**Figure 4:**
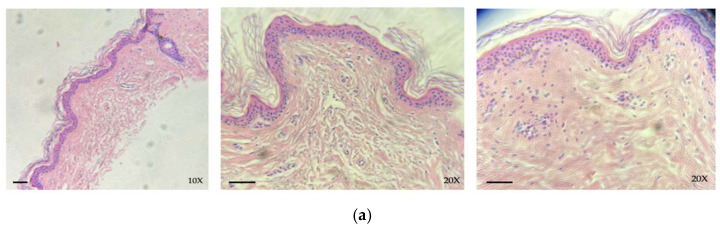

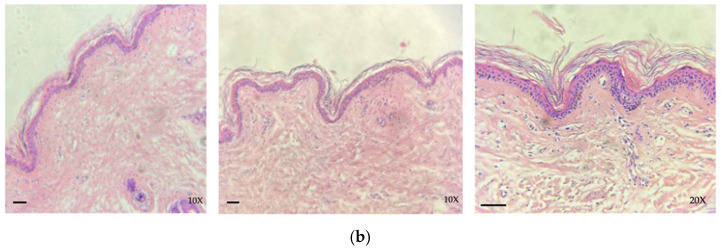
Histological evaluation of skin allograft samples before (**a**) and after (**b**) glycerol preservation: the integrity of epidermal layer and dermo-epidermal junction is preserved. (hematoxylin–eosin; scale bar: 50 µm; original magnification 10×, 20×).

Glycerol-preserved skin samples did not show any morpho-structural changes following processing. No relevant degenerative-necrotic phenomena are evident (Figure 4).

#### 3.2.3. Decellularized-Lyophilized Tissue

**Figure 5:**
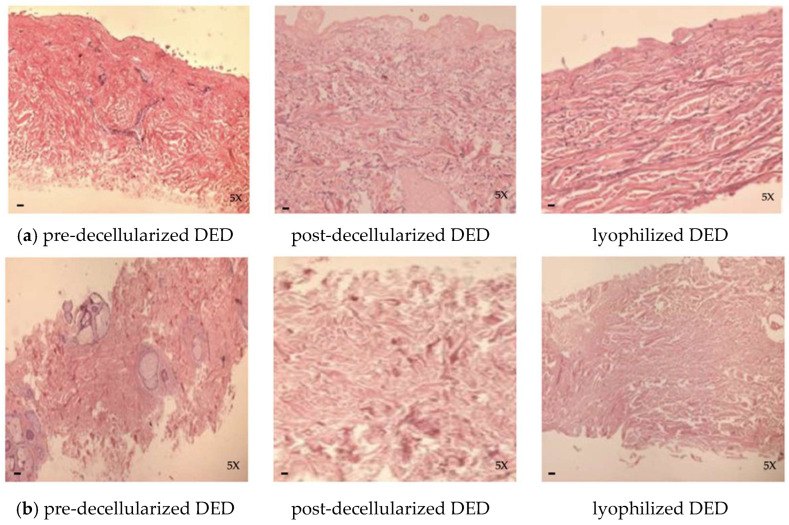
(**a**) Histological evaluation of DED and (**b**) DER samples during the various stages of processing: pre-decellularization (**left** panels), post-decellularization (**central** panels), and post-lyophilization (**right** panels) (hematoxylin-eosin; scale bar: 50 µm; original magnification 5×).

The pre-decellularized tissue, both DED and DER, shows no alterations in the morpho-structure of the elastic fibers and collagen fibers or of the skin appendages (Figure 5).

Decellularized tissue grafts, both DED and DER, showed no alterations in the morphological structure of elastic fibers and collagen fibers. The main cellular components were absent in all samples. The adnexal stroma and endothelial connective component are intact (Figure 5).

In lyophilized tissue, histological evaluation of freeze-dried DED and DER confirmed the results observed in the post-decellularization phase: the morpho-structure of tissue is preserved, and neither the collagen nor the elastic fibers appear fragmented/altered (Figure 5).

### 3.3. Immunohistochemical Investigations

#### 3.3.1. Expression of Collagen IV

**Figure 6:**
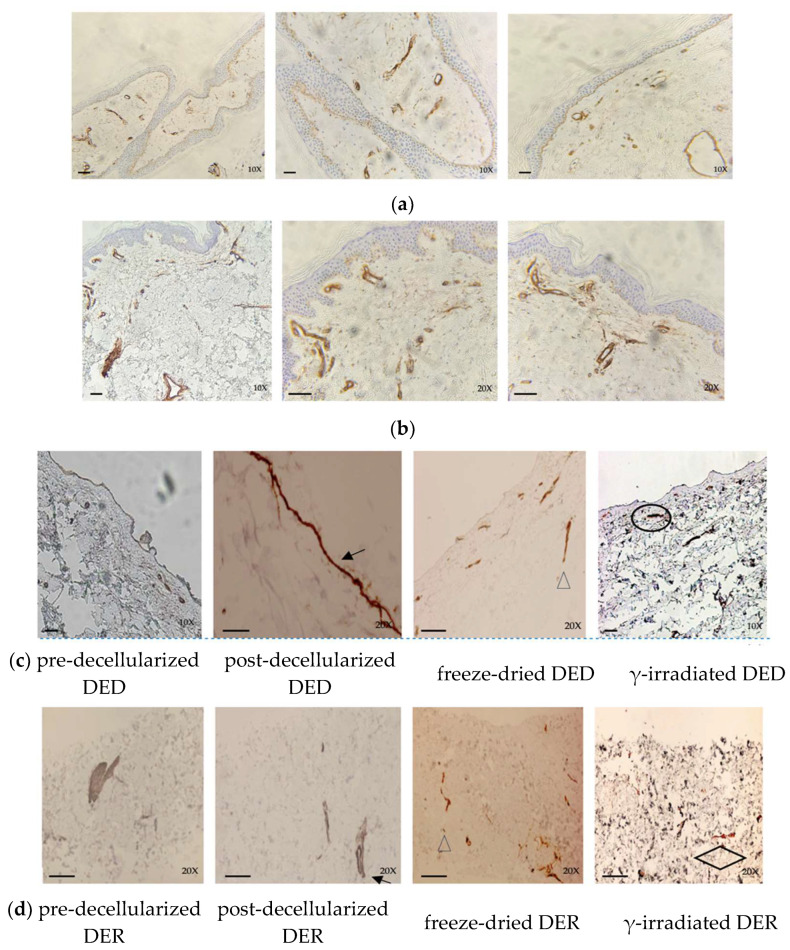
Immunohistochemical evaluation of collagen IV in cryopreserved (**a**) and glycerol-preserved tissue (**b**), pre-decellularized, post-decellularized, freeze-dried, and γ-irradiated DED samples (**c**), and pre-decellularized, post-decellularized, freeze-dried, and γ-irradiated DER samples (**d**). Residual collagen IV was observed at the level of the structural basal membrane after the decellularization process in both DER and DED samples ((**c**,**d**) black arrows) (i.e., eccrine ducts, smooth muscle cells, endothelial, and perineural basal membranes). Then, collagen IV shows only residual marking in the periadnexal basal membrane in freeze-dried DED and DER ((**c**,**d**) arrowhead/triangle), while in γ-irradiated DER it shows residual staining at the residual basement membrane and papillary dermis ((**c**) black circle) and is only sporadically arranged in tensor bundles in the deep dermis in DER samples ((**d**) black rhombus). (Abcam: anti-collagen IV antibody—(ab6586); scale bar 50 mm; 10×, 20×).

The expression of collagen IV at the level of the basement membrane, blood vessel, and periadnexal epithelium appeared to be within normal limits in both cryopreserved tissue samples (cut after rolling of the dermal graft) and glycerol-preserved tissue samples (Figure 6a,b).

In DER and DED samples taken at the end of de-decellularization, collagen IV shows residual marking at the level of the structural basal membrane after processing (i.e., eccrine ducts, smooth muscle cells, and endothelial and perineural basal membranes). In freeze-dried samples, residual collagen IV marks were observed in the periadnexal basal membrane.

Finally, in post-γ-irradiated tissue, collagen IV is normally found in the residual basement membrane of DED and papillary dermis (Figure 6c) and sporadically arranged in tensor bundles in the deep dermis (Figure 6d).

#### 3.3.2. Expression of Ki-67

**Figure 7:**
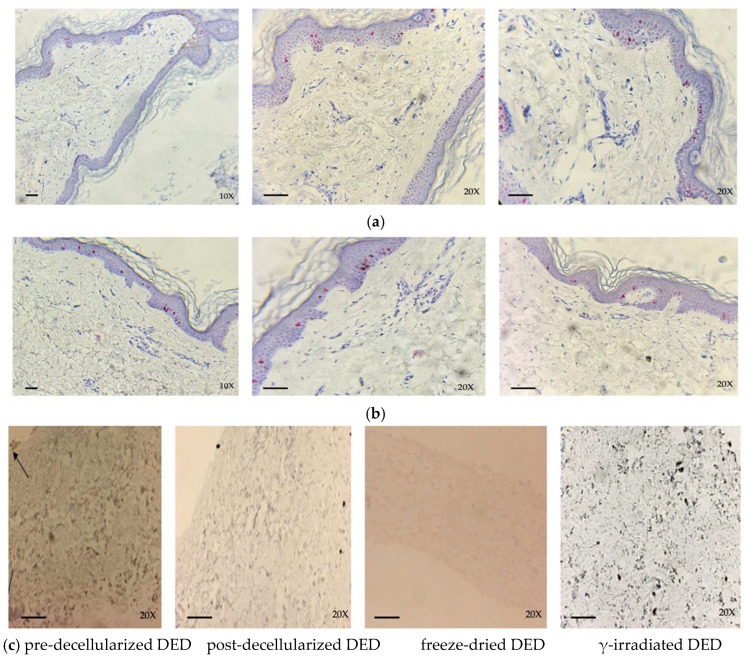

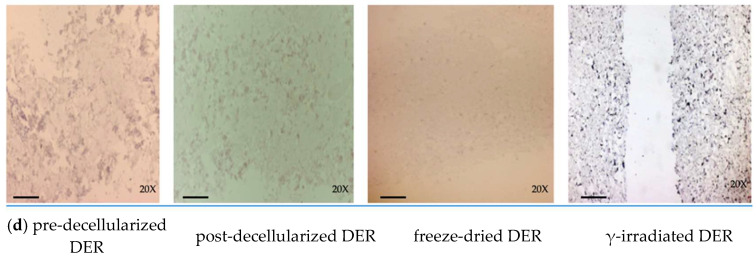
Immunohistochemical evaluation of Ki-67 in (**a**) cryopreserved skin samples and (**b**) glycerol-preserved skin samples, (**c**) in pre-decellularized, post-decellularized, freeze-dried, and γ-irradiated DED, (**d**) and in pre-decellularized, post-decellularized, freeze-dried, and γ-irradiated DER. Expression of Ki-67 ranges from weak to low in pre-decellularized DED ((**c**) black arrow) and from low to absent in pre-decellularized DER (**d**). Post-decellularization, staining was negative in DED and DER and confirmed in freeze-dried and γ-irradiated samples (**c**,**d**). (Ki-67 staining (La Roche, 790-4286); scale bar 50 mm; 10×, 20×).

Both cryopreserved and glycerol-preserved tissues showed high levels of Ki-67 at the basal and suprabasal levels, while pre-decellularized samples, as expected, show expression levels ranging from weak to low in DED and from low to absent in DER (Figure 7c,d). Post-decellularization, staining for Ki-67 was negative in DED and DER samples, ruling out the presence of residual cell viability. Moreover, these results were also confirmed by immunohistochemical evaluation performed post-lyophilization in both DED and DER. In γ-irradiated tissues, investigations show a total absence of staining in both DED and DER, indicating a lack of cells engaged in metabolic/replicative activity.

### 3.4. Clinical-Laboratory Outcomes

The clinical data concerning 13 patients managed in the hard-to-heal wound service of Siena Hospital between September 2023 and January 2024 were retrospectively analyzed, along with laboratory data related to the specific allograft employed. These patients were selected based on these criteria: having a hard-to-heal wound of clear etiology (post-traumatic, inflammatory, vascular, surgical, and burn injuries); being treated with skin/dermal allografts depending on the initial extension and depth; having assured full compliance with treatment and having attended all follow-up appointments; agreement not to use any synthetic advanced medication. Moreover, the group was recruited in a short time period in order to have a sample representative of real-life patients eligible for either simple or composite grafts with cryopreserved skin. All data are reported in Table 2 and included: (i) objective patient data (age at diagnosis, sex); (ii) lesion objective data (localization of the skin lesion on the body, dimension), (iii) lesion etiology (burns (heat and chemical), surgical dehiscence, traumatic wounds, inflammatory wounds, arterial wounds, venous wounds, and mixed vascular wounds); (iv) type of skin allograft and numbers of the specific types of skin allografts used; (v) mean skin allograft thickness, wound area, and total area of allograft used; (vi) MTT value of cryopreserved allografts; and (vii) data concerning the clinical outcome (time in days for 50% reduction of wound bed surface and time in days for complete re-epithelization of the skin lesion).

#### 3.4.1. Heat Burn Patients

**Figure 8:**
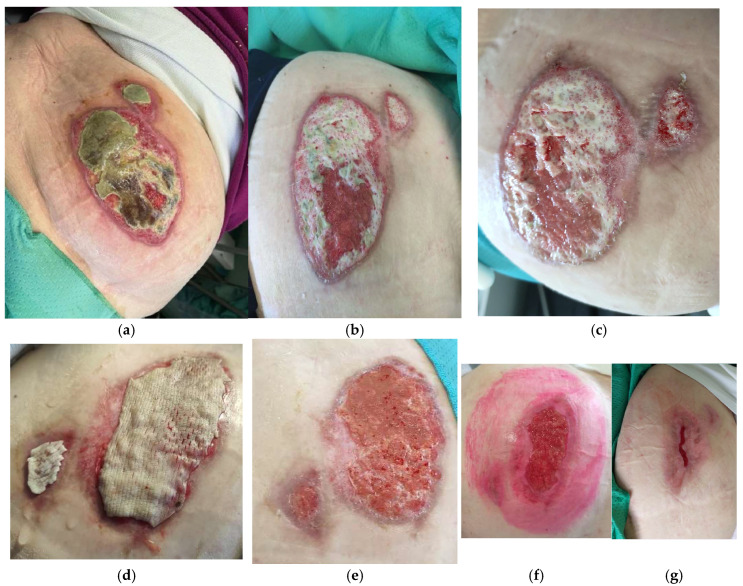
An 84-year-old woman with diabetes type I presented with a 7-day-old second-degree heat burn of the abdomen: time 0 (**a**), after mechanical enzymatic and surgical debridement (**b**,**c**) application of cryopreserved skin allograft meshed (1:3) (**d**). Clinical appearance at 1 (**e**), 3 (**f**), and 5 (**g**) weeks after grafting.

Data from three patients have been reviewed: two women (71 and 84 years old) and one man (61 years old). Localization of burns included left arm, right forearm, and hand and abdomen (Figure 8). All patients had been treated with cryopreserved skin allografts with 800 µm (two patients) or 640 µm (one patient) mean thickness, for a total amount per patient of 53 cm^2^, 111 cm^2^, and 218 cm^2^, respectively. The mean surface areas of the skin allografts were 45 cm^2^ (arm), 90 cm^2^ (arm-forearm), and 170 cm^2^ (abdomen), and the MTT values were 0.460, 0.414, and 0.414, respectively. Time expressed in days for 50% wound bed surface (WBS) reduction and for complete re-epithelization were, respectively, 10 and 21; 7 and 11; and 21 and 38. (Table 2). All three patients reported intense pain at presentation time (VAS 8, 9, 9): after the grafting procedure, pain was 2, 2, 3, and the day after 2, 1, 2.

#### 3.4.2. Chemical Burns

**Figure 9:**
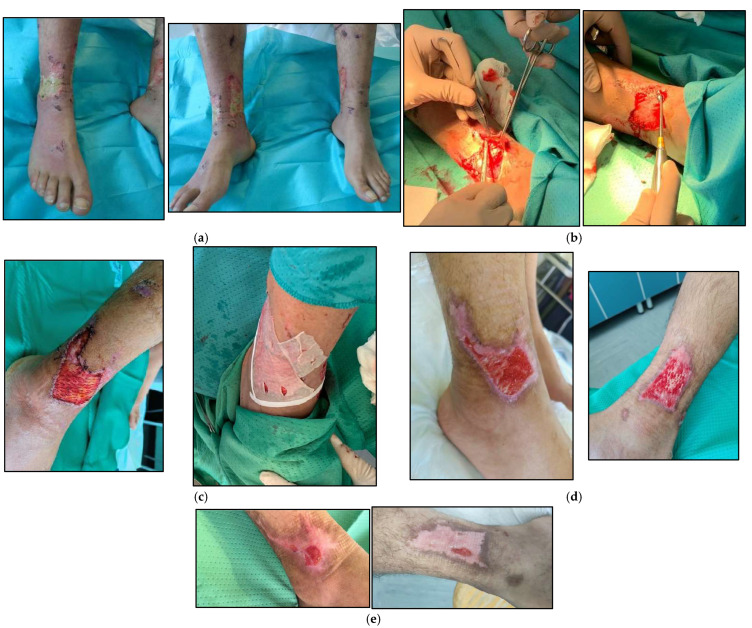
Clinical appearance of a 7-day-old chemical burn (dry cement) (**a**) in a 35-year-old male. Surgical debridement and escharotomy (**b**) performed in the dermo-surgical room, realization of the composite grafting of de-epidermized dermis (sutured with resorbable stiches) plus cryopreserved skin (**c**). Two weeks (**d**) and four weeks (**e**) after grafting, reaching 90% reduction of WBS.

An otherwise healthy 34-year old male presented with severe bilateral chemical burns involving both ankles and the third distal segment of the legs. The patient was working with quick-setting cement when the container tipped over and the cement spilled onto his legs. He presented to our HHW service after about 7 days of self-medication, with increasing pain and the high risk of developing compartment syndrome (Figure 9). The patient was hospitalized and 24 h after topical debridement underwent escharotomy in the operating room of the Dermatology Unit, with subsequent grafting of composed grafts (dermal acellular plus cryopreserved skin allograft on the right leg) and a simple graft (cryopreserved meshed skin allograft on the left leg). Overlying paraffin gauze was replaced every 5 days. The acellular dermis was completely integrated into the wound bed. The skin was replaced every 7 to 10 days. Immediate pain relief was reported. The left and right leg wounds healed after 28 and 30 days, respectively.

#### 3.4.3. Surgical Dehiscence

**Figure 10:**
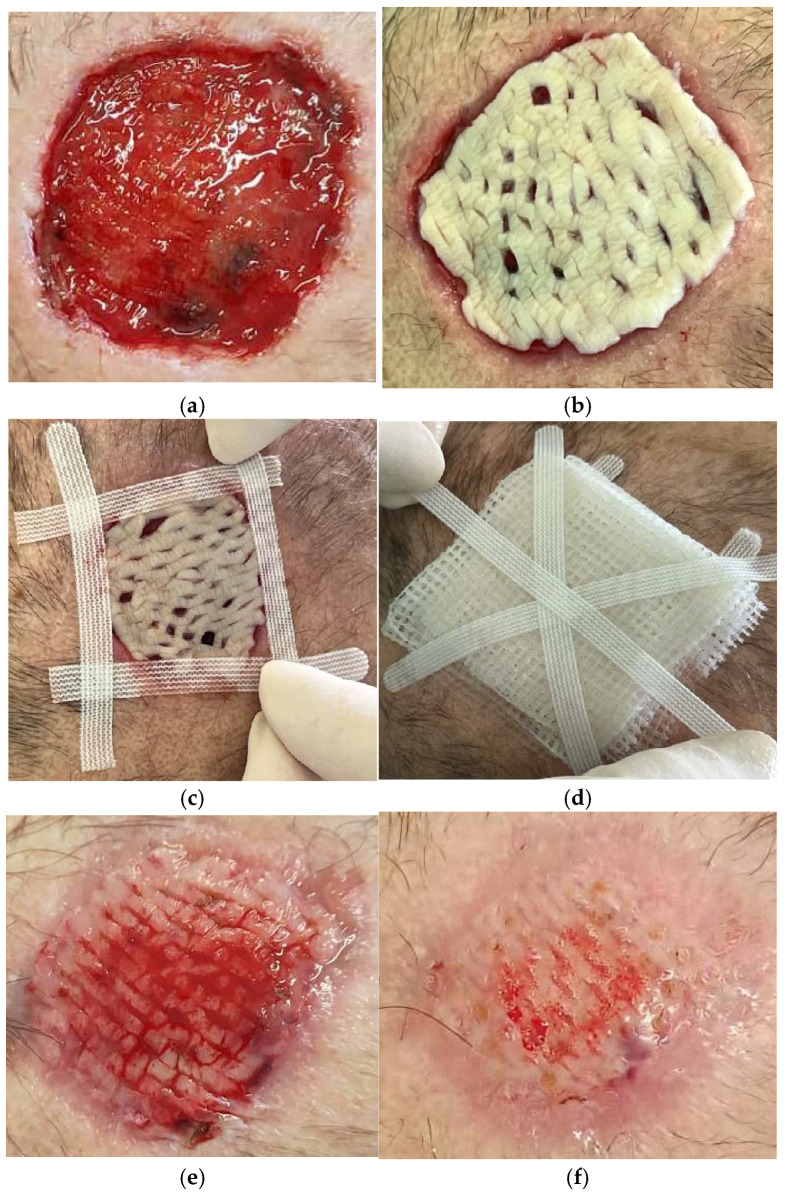
Clinical appearance after wound bed debridement (enzymatic) (**a**) of a 5.5 × 5 cm surgical dehiscence produced by surgical excision of a large basal cell carcinoma of the vertex. Application of cryopreserved meshed (1:3) skin allograft after proper shaping (**b**). Fixation of the graft (**c**) with sterile strips. Application of compressive dressing made by paraffin gauze. (**d**) Ten days after grafting, the allograft is integrated into the wound bed by 50% (**e**). Complete intake of the grafting at 20 days (**f**).

Data from one patient have been reviewed. A 66-year-old woman presented with surgical dehiscence localized to the head, due to removal of a large basocellular carcinoma (Figure 10). The patient received four cryopreserved skin allografts (total 48 cm^2^) with 800 µm mean thickness; the mean surface skin allograft area after debridement and wound bed preparation was 12 cm^2^. The MTT value was 0.467. The days needed for 50% reduction of the WBS and for complete re-epithelization were, respectively, 23 and 48 (Table 2). The pain reported by the patient disappeared immediately after grafting.

#### 3.4.4. Traumatic Wounds

**Figure 11:**
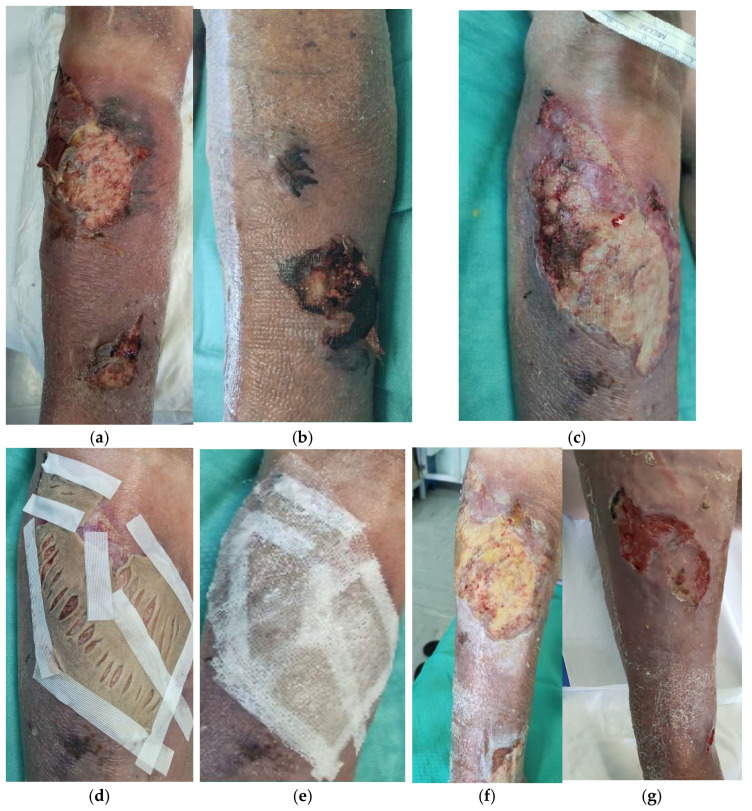
Traumatic wound in a 80-year-old man: clinical appearance at presentation time (**a,b**), 5 days after application of advanced dressings (**c**), and subsequent grafting with meshed cryopreserved skin (**d**), fixed with sterile strip and coverage with paraffin gauze (**e**). Clinical appearance one week after grafting (**f**) and healing progression 24 days after grafting (**g**).

An 80-year-old man with hypertension, diabetes type II, and hypercholesterolemia presented for multiple traumatic wounds localized on the left and right legs (Figure 11). The patient underwent three grafts of cryopreserved skin (total 237 cm^2^ of employed skin, mean thickness 600 µm, mean MTT value = 0.414) to treat a mean surface skin allograft area of 47.4 cm^2^. In total, 50% WBS reduction and complete re-epithelization were obtained after 23 and 37 days, respectively (Table 2). The patient complained of intense pain at presentation time (VAS 8) that then decreased to 1 soon after grafting.

#### 3.4.5. Inflammatory Wounds

Data from two female patients developing vasculitic lesions in the lower legs and ankle–foot were examined.

Patient 1 (aged 90 years) had a 40.2 cm^2^ mean surface wound area to treat after wound bed preparation and received four cryopreserved skin grafts and one decellularized lyophilized dermal allograft (mean thickness 640 µm, 201 cm^2^ total skin allografts). The mean MTT of the cryopreserved skin was 0.344. The patient complained of intense pain at presentation time (VAS 10) that then decreased to 0 soon after grafting.

**Figure 12:**
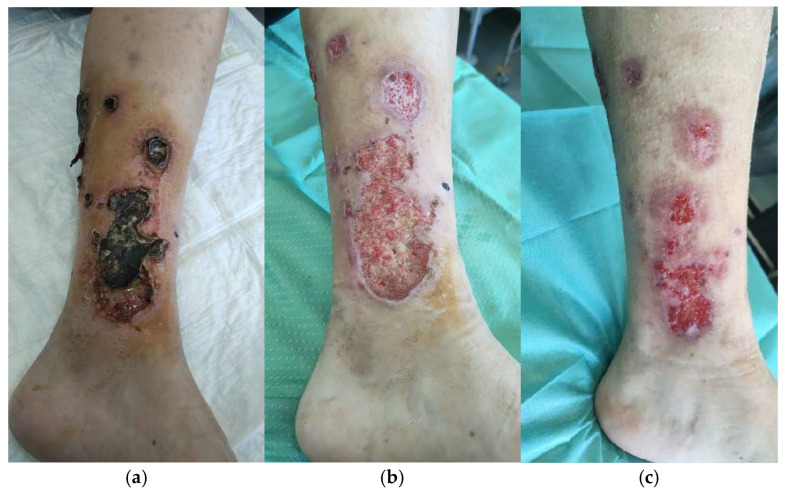
Inflammatory wound due to leukocytoclastic vasculitis of the lower leg. (**a**) Wound at first access to Dermatology Department in Siena. (**b**) Wound ready for skin allograft. (**c**) 50% WBS reduction after 33 days.

Patient 2 (a woman aged 35) developed leukocytoclastic vasculitis after pharyngeal type-b streptococcal infection and presented after 10 days of self-medication with iodine gauze (Figure 12). After adequate escharotomy, we performed multiple skin grafting with glycerol-preserved skin and glycerol-preserved decellularized dermis, having 700 µm mean thickness and 0.450 MTT, for 137 cm^2^ of total skin used. The total area to treat was 68.5 cm^2^. In total, 50% reduction of WBS and complete re-epithelization were achieved, respectively, at days 33 and 54 (Table 2).

#### 3.4.6. Vascular Wounds

We examined treatment data from six patients affected by vascular HHWs with clear-cut etiology, namely: two with arterial wounds, two with venous wounds, and two with mixed vascular wounds.

*Arterial HHWs.* Two patient exhibited lesions of the left leg with a prevalent arterial component, namely two women who were 86 and 71 years of age. The patient aged 86 years received: 13 cryopreserved skin grafts, one cryopreserved decellularized dermis graft, one glycerol-preserved skin graft, and seven decellularized lyophilized dermal allografts, for a total of 393 cm^2^ of skin area used; the mean thickness was 775 µm, and the mean MTT of the cryopreserved skin was 0.455. The mean surface of the receiving area was 65 cm^2^. In total, 50% WBS reduction and complete re-epithelization were achieved at days 51 and 82, respectively.

**Figure 13:**
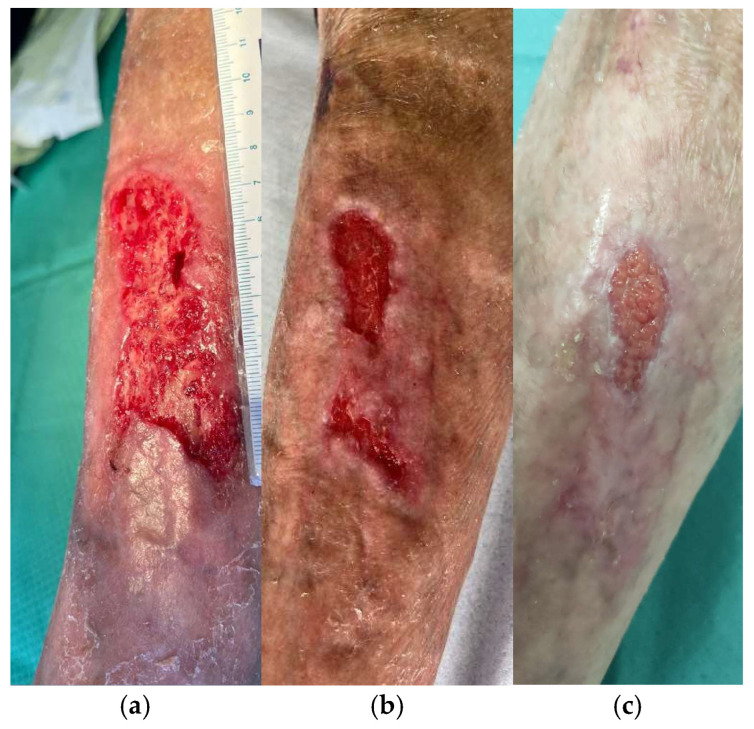
Mixed venous HHW with arterial impairment: appearance after wound bed debridement. (**a**), 3 weeks after grafting—50% WBS reduction, (**b**) and 5 weeks after grafting (**c**) obtaining a 75% WBS reduction.

The other patient (aged 71) had a 20 cm^2^ mean wound bed area and received one cryopreserved skin allograft, 800 µm thick, 20 cm^2^. The MTT of the cryopreserved skin was 0.435 (Figure 13). In total, 50% WBS reduction and complete re-epithelization were achieved at days 53 and 95, respectively (Table 2). In both cases, a significant decrease in pain was reported by both patients, namely from VAS = 8 to VAS = 2 the day after grafting and from VAS = 9 to VAS = 1 the day of grafting.

**Figure 14:**
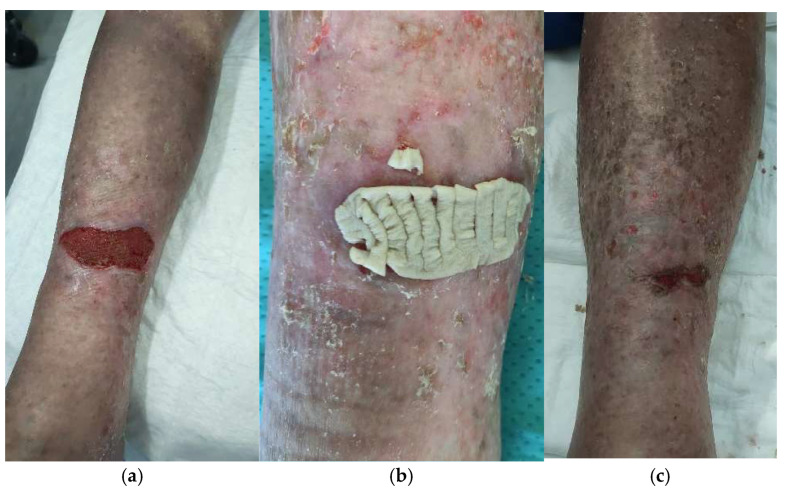
Four-month-old venous vascular wound in an 80-year-old woman, irresponsive to standard of care: clinical presentation after debridement (enzymatic and mechanical) (**a**), grafting of cryopreserved skin (**b**), and 80% WBS reduction after 2 weeks (**c**).

*Venous HHWs.* Two patients had HHWs of venous vascular origin: an 80-year-old woman and a 68-year-old man. The localization of the lesions was different: left ankle and left and right legs (Figure 14). The first patient received four glycerol-preserved dermal allografts and six cryopreserved skin allografts, 800 µm mean thickness, reaching 1005 cm^2^ of total skin area used. The MTT value was 0.462. The mean surface to treat was 100.5 cm^2^. The other patient received one cryopreserved skin allograft and one glycerol-preserved skin allograft with 800 μm mean thickness, 48 cm^2^ mean surface to cover, and 96 cm^2^ total skin used. The MTT of the cryopreserved skin was 0.322. In total, 50% WBS reduction and complete re-epithelization were obtained at 88 and 160 days in the first patient and at 76 and 110 days in the second patient, respectively (Table 2).

*Mixed HHWs.* One 72-year-old woman and one 75-year-old woman presented with mixed HHWs of the right leg.

**Figure 15:**
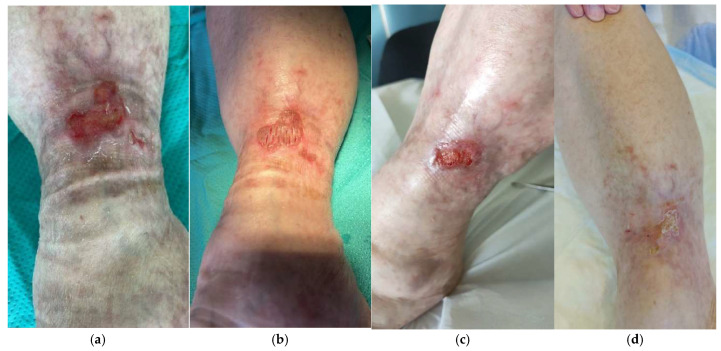
Five-month-old mixed vascular HHW in a 75-year-old woman, irresponsive to standard of care: clinical presentation after debridement (enzymatic and mechanical) (**a**), grafting of cryopreserved skin (**b**), 80% WBS reduction after 2 weeks (**c**), and complete healing after 4 weeks (**d**).

The patient aged 72 received seven glycerol-preserved and one cryopreserved skin allografts with 650 µm mean thickness and 0.480 mean MTT for the cryopreserved skin (Figure 15). Globally, 275 cm^2^ total skin was employed for a 34.4 cm^2^ mean surface to treat.

The other patient received one cryopreserved skin graft, four glycerol-preserved skin grafts, two glycerol-preserved decellularized dermis grafts, and three lyophilized decellularized dermal allografts with 740 µm mean thickness: 369 cm^2^ total skin was used to treat a total of 36.9 cm^2^. The MTT of the cryopreserved skin was 0.399.

In total, 50% WBS reduction and complete re-epithelization were achieved at days 53 and 112, respectively, in the first patient and days 31 and 77, respectively, in the second patient (Table 2).

## 4. Discussion

**Figure 16:**
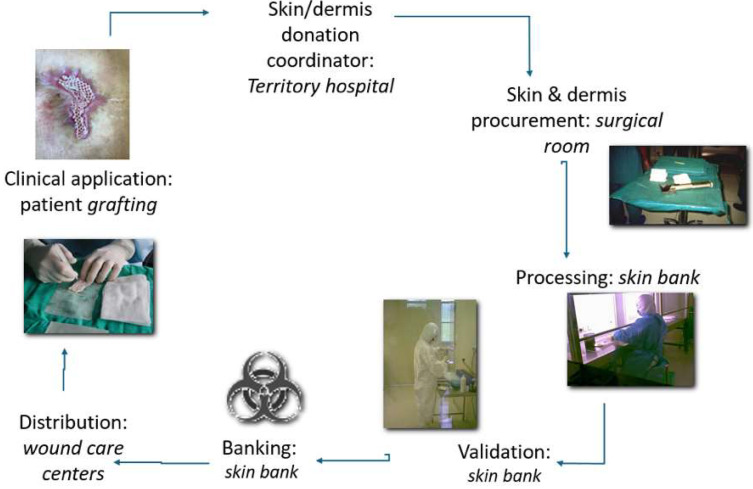
Schematic representation of the skin tissue procurement–application virtuous circle: a sustainable approach.

The existence of skin banks is basically supported by the request for skin allografts/bank bioproducts, and, in parallel, skin bank activity is strictly integrated into a complex network linking skin donors and skin recipients [47,48,49] (Figure 16). Tissue establishments, although they can perform different preservation and storage procedures, share the necessity to guarantee reproducibility, as well as the use of standardized methods, clear traceability, and safety in all phases of processing, in order to respect current regulations and avoid any biological contamination or risk of disease transmission [50,51,52,53,54]. For this purpose, periodic revision of standard operating procedures, along with continuous training and improvement of quality objectives, helps maintain a high level of attention and responsibility among skin bank operators and guarantee progressive quality improvement [13,22,50].

The demand for skin bank bioproducts is strictly related to clinical practice in hospital and in outpatient settings and takes into account the individual experience of surgeons in treating wounds with tissue allografts [40,55]. Despite their undoubted efficacy, a large number of physicians (vulnologists, plastic surgeons, dermatologists, vascular surgeons, orthopedics, maxillo-facial surgeons, etc.) are not aware of this therapeutic option [56,57,58,59].

Last but not least, skin bank allografts have to face every day more “competitors” in the aggressive market of wound healing products, such as semi-synthetic or synthetic skin substitutes. However, only skin allografts from cadaver donors can be defined as “skin equivalent” since they are the only products that fully reproduce the lost substance. They represent the gold standard for HHWs and in general are beneficial also for superficial wounds because they induce dermal and skin regeneration, rather than scarring [38,39,40,41,42,43,44,45,46] due to the regenerative properties of cryopreserved skin and dermis, which significantly reduces the closure time.

There is a literature consensus that the clinical benefits in terms of elasticity and quality of the regenerated tissue, pain control, and rapidity of healing are superior, especially in HHWs of patients with systemic comorbidities (hypertension, diabetes, Cushing syndrome, inflammatory diseases, etc.) lead to choosing skin allografts as a therapeutic option.

In burns, surgical dehiscence, and trauma, cryopreserved tissue is important because cells remain vital, as assessed by MTT. Applying vital tissue provides a significative stimulus for burn repair. Moreover, application of cryopreserved allografts obtains excellent results in terms of rapidity of healing also in other HHWs, such as post-traumatic or surgical HHWs and some inflammatory wounds (e.g., ulcers from spider/leishmania bites). This can be explained not only by the regenerative stimulus on cytokine pathways given by the presence of residual viable cells in the graft, but also by the higher adherence properties showed by cryopreserved allografts compared to glycerolized ones. This results in a more stable dressing in general when the patient goes home. In addition, cryopreserved allografts are more easily meshed, resized, and shaped according to the wound characteristics, compared to glycerol-preserved allografts [9,10,11,20,29,30,31,38,39].

In vascular lesions and some inflammatory lesions (e.g., pyoderma gangrenosum), the approach is different, as the wounds are often deep, so they require the application of dermal allografts, which act by replacing the injured dermis. In the case of painful ulcers, glycerol-preserved allografts are preferred due to their antalgic properties, calming the signaling of pain fibers. It is widely known that wounds need to be prepared before the application of allografts, by eliminating possible fibrin and infections. When infections are resistant and cannot be fully eliminated before grafting, glycerol-preserved allografts can be used, as glycerol itself has antimicrobial activity [20,38,39].

Concerning the clinical outcome of the 13 patients here considered, we globally observed wound closure in all cases, reduced time for the re-epithelization phase, shortening of the whole healing time, protection of deep subcutaneous structures, results maintenance over time, and no keloid formation. Globally evaluating the outcome of the different HHW types here examined from the patients’ point of view, there was a consensus on pain reduction (expressed as a relevant decrease in VAS scale score) soon after grafting, global satisfaction for the dressing maintenance (that required usually one change every 7–10 days performed in the HHW service of the hospital), final healing occurring previously than expected, happiness with the aesthetic and functional outcome, and global improvement of quality of life.

Specific studies comparing the clinical outcome derived from the use of skin allografts versus that of synthetic commercial equivalents are few: this is due to the difficulty in standardizing lesions and patients, as each wound is intrinsically different from another, and the patient profile as well. The majority of available data to date still support the superiority of skin and dermal allografts [40,60,61,62]. It is worth mentioning that there have been reports of hypertrophic scarring after grafting, but this is essentially related to the specific wound and dressing technique characteristics, as well as to the patient profile [63].

Finally, synthetic commercialized skin equivalents are often expensive and have a major impact on national health systems [64,65]. In contrast, while the cost of procuring and applying donor skin is very low and can be fully reimbursed by the healthcare systems, it is considered a tissue donation. Indeed, skin banks in Europe are not authorized to sell skin and dermis samples donated from human cadavers, resulting in a sustainable approach. Another scenario is that of the United States, where dermis or epidermis plus dermis samples obtained from cadavers are processed and sold by companies, along with umbilical cords. Therefore, the accessibility of bank bioproducts and greater economic viability make allografts preferable, especially in cases of chronic wounds requiring continuous dressing [27,40,41,42]. Actually, skin bank bioproducts/allografts represent a sustainable therapeutic approach, based on the “virtuous circle” involving organ/tissue donation coordinators, retrieval teams, personnel from tissue establishments, physicians, and end-users (patients) (Figure 16).

It should be noted that, to date, there are not enough data/specific clinical trials/comparative studies for skin allografts and recently introduced advanced wound care technologies including bioengineered skin substitutes enriched with specific growth factors/cytokines [66,67].

On the other hand, the application of extended skin grafting plus cultured epithelial autografts provided encouraging results [68]. This research is extremely promising, and the possibility of creating a skin equivalent that is “richer” in stimulating healing factors or better designed structurally is a matter of research (e.g., 3D-printed). Nevertheless, it remains to be seen in the future if the costs for these products could be afforded by national health systems and applied on a large scale to public hospital patients.

Study limitations include: (i) selection bias due to the fact that patients were recruited from a HHW service where the application of allograft as a healing technique is very easy to be realized exactly at the right time (i.e., prepared wound bed), compared with medical centers far from skin bank establishments; (ii) the fact that in our HHW service we could choose between a great variety of skin allografts (cryopreserved/glycerol-preserved skin/DED, lyophilized acellular DED/DER), which is not so common in other tissue banks; (iii) patients/lesions were retrospectively selected and thus were eligible according to allograft application (e.g., patients not allergic to penicillin, lesion not of neoplastic nature, lesion not under infectious process); and (iv) finally, the experience of physicians in this healing technique field should be taken into consideration.

## 5. Conclusions

Skin allografts are deemed effective in the treatment of many kinds of skin loss and may be life-saving. They have important clinical uses, behaving as physiological medication, promoting wound healing, shortening hospitalization time, controlling pain, and protecting dermal and subcutaneous structures (cartilage, tendons, nerves, and bones).

They are also successfully used as skin substitutes that incorporate the dermal component into the wound bed, guiding a more physiological healing process. Skin bank bioproducts can be regarded as an almost fully sustainable therapeutic approach, based on a circular system that involves organ/tissue donation coordinators, retrieval teams, personnel from the tissue establishments, physicians, and patients.

## Figures and Tables

**Table 1:** Comparison of viability assessment (MTT assay) measurements of skin allograft before and after 15 days of cryopreservation, expressed as optical density (OD570nm). Data from viability assessment (MTT assay) of skin allograft before and after 15 days of cryopreservation as optical density (OD_570nm_).

OD_570_ Residual			
	Pre	Post	Residual Vitality (%)
Sample 1	0.644	0.444	69
Sample 2	0.952	0.564	59
Sample 3	0.6	0.3	50
Sample 4	1.145	0.563	49
Sample 5	0.426	0.207	49
Sample 6	0.727	0.283	39
Sample 7	0.509	0.319	63
Sample 8	1.496	0.374	25
Sample 9	1.285	0.47	37
Sample 10	0.747	0.411	55
Sample 11	0.943	0.618	66
Sample 12	0.901	0.448	50
Sample 13	1.003	0.627	63
Mean value	0.875 ± 3.11	0.433 ± 0.134	50

**Table 2:** Clinical characteristics of patients with hard-to-heal wounds (HHWs), laboratory details of the various allografts employed, and clinical outcome measures including wound bed surface (WBS) reduction and healing time.

Patient Information			Skin Graft Data						Clinical Outcome	
Age (Years)	Sex	Localization	Type of Skin Graft	N° Grafts	Thickness (Mean Value in μm)	Area (Mean Value in cm^2^)	Total cm^2^ Skin Used (cm)	MTT	Days for 50% WBS	Days for Healing
**Heat burns**										
71	F	Left arm	Cryopreserved skin	2	800	45	53	0.460	10	21
61	M	Right forearm and hand	Cryopreserved skin	3	800	90	111	0.414	7	11
84	F	Abdomen	Cryopreserved skin	5	640	170	218	0.414	21	38
**Chemical burn**										
34	M	Legs	Decellularized lyophilized dermis	1	640	130	140			30
			Cryopreserved skin	2	600	300	362	0.440		30
**Surgical dehiscence**										
66	F	Head	Cryopreserved skin	4	800	12	48	0.467	23	48
**Traumatic wound**										
80	M	Left and right legs	Cryopreserved skin	3	600	47.4	237	0.414	23	37
**Inflammatory wounds**										
93	F	Right foot	Decellularized lyophilized dermis	1	640	40.2	201		33	54
			Cryopreserved skin	4				0.344		
35	F	Left leg	Cryopreserved skin	1	700	68.5	137	0.450	8	13
			Glycerol-preserved decellularized dermis	1						
**Arterial wounds**										
86	F	Left leg	Decellularized lyophilized dermis	7	775	16.4	393		51	82
			Cryopreserved decellularized dermis	1						
			Cryopreserved skin	13				0.455		
			Glycerol-preserved skin	1						
71	F	Left leg	Cryopreserved skin	1	800	20	20	0.435	53	95
**Venous wounds**										
80	F	Left ankle	Glycerol-preserved decellularized dermis	4	800	100.5	1005		88	160
			Cryopreserved skin	6				0.462		
68	M	Left and right legs	Glycerol-preserved skin	1	800	48	96		76	110
			Cryopreserved skin	1				0.462		
**Mixed vascular wounds**										
72	F	Left leg	Cryopreserved skin	1	650	34.4	275	0.480	53	112
			Glycerol-preserved skin	7						
75	F	Left leg	Cryopreserved skin	1	740	36.9	369	0.399	31	77
			Glycerol-preserved skin	4						
			Glycerol-preserved decellularized dermis	2						
			Lyophilized decellularized dermis	3						

## Data Availability

The raw data supporting the conclusions of this article will be made available by the authors on request.
